# HuR-Targeted Small Molecules Reduce *Pseudomonas aeruginosa* Adhesion in Cystic Fibrosis Airway Epithelial Cells

**DOI:** 10.3390/ijms27010232

**Published:** 2025-12-25

**Authors:** Roberta Listro, Angelica Pellegrini, Giacomo Rossino, Pasquale Linciano, Giampiero Pietrocola, Simona Collina

**Affiliations:** 1 Department of Drug Science, University of Pavia, Via Taramelli 12, 27100 Pavia, Italy; roberta.listro@unipv.it (R.L.); giacomo.rossino@unipv.it (G.R.); pasquale.linciano@unipv.it (P.L.); 2 Department of Molecular Medicine, University of Pavia, Via Taramelli 3B, 27100 Pavia, Italy; angelica.pellegrini@unipv.it

**Keywords:** RNA binding protein, HuR, *Pseudomonas aeruginosa*, cystic fibrosis

## Abstract

Antibiotic-resistant infections remain a major challenge in cystic fibrosis (CF), where chronic *Pseudomonas aeruginosa* colonization drives lung infection. The overexpression of adhesion-related proteins and extracellular matrix components, including fibronectin (Fn), facilitates bacterial colonization. Recent evidence identifies the RNA-binding protein Human Antigen R (HuR) as a key regulator of this process, as it stabilizes *Vav3* mRNA, promoting Fn deposition and the formation of bacterial docking platforms. Here, we report the synthesis, optimization, and functional evaluation of the HuR-targeted small-molecule (*2S,3S*)-**BOPC1**. Functional assays in CF human airway epithelial cells demonstrated that (*2S,3S*)-**BOPC1** significantly reduced *P. aeruginosa* adhesion in a dose-dependent manner without detectable cytotoxic effects. These findings provide the first evidence that targeting HuR can disrupt the HuR–*Vav3*–Fn axis, reducing bacterial attachment. This host-directed approach represents a promising strategy to prevent chronic infections in CF without promoting antibiotic resistance.

## 1. Introduction

Infections caused by antibiotic-resistant bacteria have emerged as one of the most pressing global health challenges, with an estimated 1.27 million deaths annually [1]. This number is expected to rise dramatically, with projections indicating up to 10 million deaths per year by 2050, surpassing cancer-related mortality [2]. Among the most vulnerable populations are individuals with chronic diseases or immunocompromised conditions, including patients with cystic fibrosis (CF), who are particularly susceptible to recurrent bacterial colonization and chronic infections.

Cystic fibrosis is a life-shortening autosomal recessive disorder caused by mutations in the *CFTR* (cystic fibrosis transmembrane conductance regulator) gene, which encodes a cAMP-regulated chloride and bicarbonate channel located on the apical surface of epithelial cells [3]. Defective CFTR function leads to dysregulated ion transport and abnormal hydration of the airway surface liquid, resulting in thick, viscous mucus and impaired mucociliary clearance [4]. Such dehydrated mucus hampers efficient pathogen removal, promoting an environment that favors bacterial growth and biofilm formation [5,6]. In addition, CF airways exhibit chronic inflammation that begins early in life and progressively damages lung tissue. Elevated levels of cytokines such as IL-8, IL-6, and TNF-α sustain neutrophil activation, leading to protease release, oxidative stress, and epithelial barrier disruption [7]. This inflammatory milieu establishes a self-perpetuating cycle of infection and inflammation.

Despite significant advances in CF management, including the advent of CFTR modulators (e.g., ivacaftor, lumacaftor, tezacaftor, and elexacaftor), chronic lung infections remain a major clinical challenge [8]. Many patients continue to suffer from recurrent infections dominated by multidrug-resistant pathogens, primarily *Pseudomonas aeruginosa* [9].

Indeed, *P. aeruginosa* represents the major opportunistic pathogen in CF lungs. Up to 80–90% of adults with CF in some European regions are chronically infected with *P. aeruginosa*, which drives progressive pulmonary decline through biofilm formation and the secretion of virulence factors [10]. Current treatments rely on antibiotics such as ceftazidime, ciprofloxacin, tobramycin, and colistin [11,12]. However, the emergence of multidrug-resistant (MDR) strains has drastically reduced therapeutic efficacy [13].

The growing prevalence of antibiotic resistance and the limited pipeline of new antimicrobial agents underscore the urgent need for alternative strategies. One promising approach is to interfere with the host–pathogen interaction, specifically by targeting bacterial adhesion and colonization mechanisms. In CF airways, the overexpression of adhesion-related proteins promotes the deposition of fibronectin (Fn), a key extracellular matrix component that facilitates the attachment of pathogens such as *P. aeruginosa* to epithelial surfaces [14]. This process is further enhanced by the upregulation of Vav3, a guanine nucleotide exchange factor that modulates cytoskeletal dynamics, cell adhesion, and migration [15].

**Figure 1:**
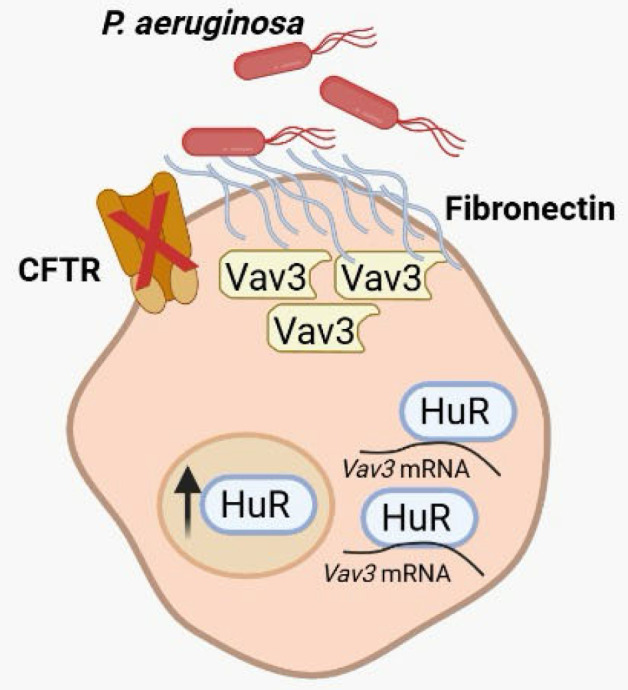
Schematic representation of fibronectin overexpression in cystic fibrosis (CF) airway epithelial cells. CFTR dysfunction (indicated by the red “X”) leads to increased expression of the RNA-binding protein HuR (black arrow), which in turn enhances fibronectin expression. Elevated fibronectin levels promote increased adhesion of bacteria, such as *P. aeruginosa*, to the airway epithelium.

Vav3 expression is post-transcriptionally regulated by Human Antigen R (HuR), an RNA-binding protein (RBP) belonging to the ELAV family, making it a key upstream regulator of this adhesion pathway [16]. HuR is ubiquitously expressed and plays a crucial role in the stabilization and translation of mRNAs containing AU-rich elements (AREs) in their 3′-untranslated regions [17,18]. Through this mechanism, HuR regulates proteins involved in cell proliferation, stress response, inflammation, and immune regulation (Figure 1) [19,20].

In the CF context, HuR has emerged as a pivotal regulator of epithelial homeostasis and inflammatory signaling. Several studies have demonstrated that HuR expression and cytoplasmic translocation are increased under inflammatory and oxidative stress conditions, such as the environment of CF airways. Moreover, by stabilizing Vav3 mRNA, HuR contributes to the formation of Fn-enriched adhesion sites that facilitate bacterial colonization [16].

Targeting HuR offers an innovative therapeutic opportunity to mitigate bacterial adhesion and infections without directly exerting selective pressure on pathogens. This host-directed approach may circumvent the development of antibiotic resistance while addressing a critical step in the infection process. Based on this rationale, we investigated whether targeting HuR could modulate bacterial adhesion and infection in CF airway epithelial cells. Here, we report the optimization of the hit compound **BOPC1** and demonstrate its efficacy against *P. aeruginosa* infection in CF models.

## 2. Results

Vav3 overexpression promotes the formation of Fn-rich adhesion sites that facilitate the attachment of pathogens such as *P. aeruginosa* to the CF airway epithelium [21]. This overexpression results from increased mRNA stability mediated by HuR, which binds to and stabilizes Vav3 transcripts, thus enhancing their translation in CF cells [16]. Given the central role of HuR in controlling Vav3 expression and, consequently, bacterial adhesion, targeting the HuR–mRNA interaction represents a promising strategy to limit pathogen colonization in CF airways.

To explore this hypothesis, we developed small-molecule modulators of HuR–mRNA interaction. Among the compounds identified in our previous screening campaign [22], **BOPC1** emerged as a promising HuR binder. Building on structure-activity relationship (SAR) studies and STD-NMR data indicating preferential binding of the (S)-configured enantiomer, we optimized its synthesis to obtain the enantiomerically pure (+)-(*2S,3S*)-**BOPC1**, aiming to evaluate its biological activity and efficacy in CF infection models.

### 2.1. Preparation of Enantiomerically Pure (+)-(2S,3S)-BOPC1

**Scheme 1:**
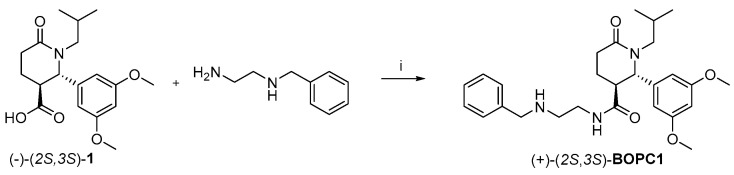
Synthesis of (+)-(*2S,3S*)-**BOPC1**. (i) (−)-(*2S,3S*)-**1** (1 quiv.), benzyl ethylenediamine (1 equiv.), B(OCH_3_)_3_ (2 equiv.), MeCN, MW, 3 × 5 min, 250 Watt. 250 PSI, 110 °C.

Previous STD-NMR studies demonstrated that the (S)-enantiomer of **BOPC1** displays stronger binding affinity toward HuR than the (R)-enantiomer [22]. The interaction is primarily mediated by the 3,4-dimethoxybenzoyl moiety, which was designed to mimic nucleosides U8 and U9 within the RNA recognition motif, the region known to establish the most extensive HuR-RNA interactions. To obtain a suitable amount of (*2S,3S*)-**BOPC1** required for this study, we initially directed our efforts toward its preparation, as outlined in Scheme 1.

Starting from enantiopure (−)-(*2S,3S*)-**1**, previously prepared and fully characterized in our laboratory [22], a microwave (mw) assisted coupling reaction was performed using B(OCH_3_)_3_ as coupling reagent in acetonitrile (MeCN). After 3 cycles of 5 min, the crude product was treated with Amberlite resin IRA734 to scavenge residual boron reagent. The mixture was washed with a saturated NaHCO_3_ solution to remove the excess of acid **1**, and the final product was isolated by silica gel filtration.

The stereochemical integrity was confirmed by HPLC using a Chiralpak IC column (mobile phase: *n*-hexane/IPA/DEA 75:25:0.1; flow rate: 1 mL/min), following the analytical method previously established by our group [22]. The reaction of (−)-(2S,3S)-**1** $([{\alpha ]}_{D}^{20}=$ −49.4°, *c* = 1% in CHCl_3_, *e.e.* = 99.9%) with benzyl ethylenediamine afforded a single enantiomer with an enantiomeric excess of 99.9% $[{\alpha ]}_{D}^{20}=$ (+74.0 c = 0.05% in MeOH), corresponding to (+)-(*2S,3S*)-**BOPC1**.

Overall, the new synthetic strategy enabled improvements in terms of efficiency and sustainability. Performing the resolution step upstream minimizes the loss of unwanted enantiomeric material, thereby reducing the environmental impact factor (E-factor) [23]. Moreover, the quick and energy-effective MW-assisted condensation of enantiopure (−)-(*2S,3S*)-**1** with benzyl ethylenediamine was performed in ACN as a less hazardous and problematic alternative to DMF. The downstream purification was also improved thanks to the use of recyclable Amberlite resin and a rapid filtration on silica gel.

### 2.2. Inhibition of P. aeruginosa Adhesion to CF Airway Epithelial Cells

To evaluate whether (+)-(*2S,3S*)-BOPC1 could modulate bacterial adhesion, we employed differentiated human airway epithelial cells (HAECs) derived from CF donors homozygous for the F508del-CFTR mutation. Differentiated HAECs were cultured at air-liquid interface (ALI) and pre-treated with (+)-(*2S,3S*)-BOPC1 at 50 µM and 100 µM for 24 h prior to apical infection with *P. aeruginosa* strain PAO1 (1 × 10^7^ CFU). Following 1 h of infection at 37 °C and 5% CO_2_, adherent bacteria were quantified by lysing cells and enumerating colonies.

**Figure 2:**
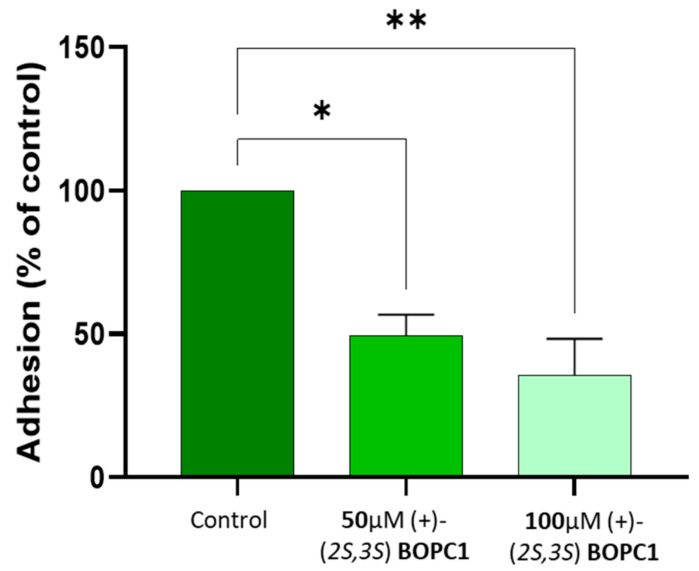
Validation of BOPC1 Anti-Adhesive Properties in CF Cells. Following overnight incubation with (+)-(*2S,3S*)-BOPC1, HAECs from FC patients were infected apically with *P. aeruginosa* cells. One hour after infection, adherent bacteria were enumerated by plating serial dilutions of the infected cells, detached from the transwell with Triton X-100 and repeated pipetting. The percentage of bacterial adhesion was calculated relative to the initial inoculum and was normalized to the adhesion of the control (vehicle-treated) strain, set at 100%. Data reported represent the mean of two independent experiments, each performed in duplicate. Statistically significant differences are indicated. * *p* < 0.05, *n* = 2, 49.50 ± 7.17; ** p < 0.01, *n* = 2, 35.72 ± 12.55 (One-way ANOVA).

Treatment with (+)-(*2S,3S*)-**BOPC1** significantly reduced *P. aeruginosa* adhesion in a dose-dependent manner (Figure 2). At 50 µM, bacterial adhesion was reduced by approximately 50% compared to vehicle-treated controls, while 100 µM treatment resulted in a 65% reduction. Importantly, treatment at both concentrations did not elicit any detectable cytotoxic effects, as evidenced by the maintenance of typical epithelial cell morphology, uniform confluence, and overall structural integrity of the epithelium, as assessed through visual examination under phase-contrast light microscopy.

## 3. Discussion

Taken together, these findings provide the first direct evidence that pharmacological modulation of HuR can effectively impair *P. aeruginosa* adhesion to CF airway epithelial cells. The observed dose-dependent reduction in bacterial colonization is consistent with potential interference of (+)-(*2S,3S*)-**BOPC1** with the HuR–*Vav3*–fibronectin axis, leading to reduced formation of adhesion sites permissive for bacterial attachment. While existing evidence supports the proposed mechanism, it remains hypothesis-driven. Therefore, further investigations will be required to confirm target engagement, including analyses of HuR localization, Vav3 mRNA stability, and fibronectin deposition. Moreover, future studies should discriminate between indirect host-mediated effects and potential direct interference with bacterial adhesion to HAEC epithelia.

Several aspects of these results deserve attention. First, the magnitude of adhesion inhibition (approximately 65% at 100 µM) suggests that HuR represents an important regulatory node in this pathway, and the additional mechanisms may contribute to bacterial adhesion in CF airways. Future studies should investigate whether a multitarget approach, i.e., simultaneously targeting HuR and other adhesion-related factors, could achieve more complete inhibition.

Second, the lack of cytotoxicity at therapeutic concentrations is encouraging and distinguishes this approach from conventional antibiotics that often cause collateral damage to host tissues. However, more comprehensive toxicity assessments, including long-term ALI culture studies and evaluation of epithelial barrier function markers (e.g., tight junction proteins), are necessary to fully characterize the safety profile.

**Figure 3:**
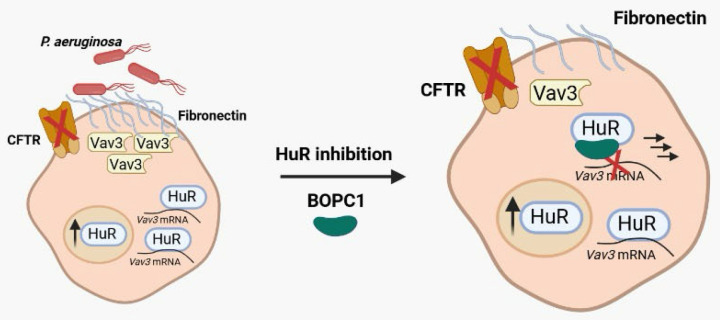
Schematic representation showing how HuR inhibition attenuates fibronectin overexpression in cystic fibrosis (CF) airway epithelial cells. In CF patients, CFTR dysfunction (red “X”) is associated with HuR overexpression. Inhibition of the HuR–Vav3 mRNA complex (red “X”) activates a series of downstream molecular mechanisms (represented by the three arrows), ultimately reducing fibronectin overexpression, limiting the formation of luminal fibronectin docking platforms, and decreasing *P. aeruginosa* adhesion.

Lastly, while our data demonstrates a clear functional activity in primary CF cells, the mechanism by which (+)-(2S,3S)-**BOPC1** disrupts HuR function remains to be clarified (Figure 3). Direct binding studies (e.g., surface plasmon resonance, isothermal titration calorimetry), together with the assessment of HuR subcellular localization and the quantification of Vav3 and Fn levels, would strengthen the mechanistic rationale and confirm target engagement.

Nonetheless, these results establish, for the first time, the therapeutic potential of HuR-targeted small molecules as host-directed agents against *P. aeruginosa* infection, and support the development of (*2S,3S*)-**BOPC** derivatives for preventing or reducing bacterial colonization in CF lungs. In this perspective, in-depth biochemical and structural studies aimed at elucidating the inhibition mechanism are ongoing, and a small library of enantiopure analogues will be developed to assess their affinity toward the target, their ability to inhibit Fn formation, and consequently bacterial adhesion.

## 4. Materials and Methods

### 4.1. General

All reagents and solvents employed for the synthesis were obtained from commercial suppliers (Sigma-Aldrich, Milano, Italy; VWR) and used without further purification unless otherwise stated. Reaction progress was monitored by thin-layer chromatography (TLC) on silica gel 60 F254 aluminum-backed plates (Merck, Fluka Kieselgel, Darmstadt, Germany). Spots were visualized under ultraviolet (UV) light and by ninhydrin staining when appropriate. Microwave-assisted reactions were carried out using a Discover^®^ SP single-mode microwave reactor (CEM Corporation, Matthews, NC, USA). Nuclear Magnetic Resonance (NMR) spectra were recorded on two different Bruker spectrometers: an Avance 400 (^1^H at 400.134 MHz, ^13^C at 100.62 MHz) and an Avance Neo 700 (^13^C at 176 MHz). Specific optical rotations were measured using a JASCO DIP-100 polarimeter (JASCO Corporation, Tokyo, Japan) fitted with sodium (λ = 589 nm) and mercury (λ = 405 nm) lamps. Analytical HPLC analyses were carried out on a JASCO chromatographic system equipped with a PU-1580 pump, DG-2080-53 degasser, LG-1580-02 mixer, and an MD-1510 photodiode array detector. Sample injections were performed using a Rheodyne 7125 valve (Rheodyne, Rohnert Park, CA, USA) fitted with a 10 μL loop. Semi-preparative HPLC experiments were performed on the same system, using a 1 mL injection loop. All chromatographic-grade solvents (*n*-hexane and isopropanol) were obtained from VWR.

### 4.2. Synthetic Procedure

#### N-(2-(Benzylamino)ethyl)-2-(3,5-dimethoxyphenyl)-1-isobutyl-6-oxopiperidine-3-carboxamide

To a solution of acid-**1** (98 mg, 0.30 mmol) dissolved in MeCN (3 mL), B(OCH_3_)_3_ (62.4 mg, 0.6 mmol) and *N*-benzylethylendiamine (0.045 mL, 0.30 mmol) were sequentially added. The reaction mixture was conducted with MW through 3 cycles of five minutes, using a ramp time of 3 min, 250 Watt, 250 PSI, high stirring at 110 °C. The reaction mixture was concentrated in vacuo, dissolved in MeOH (4 mL), and IRA734 was added to remove the coupling reagent using mechanical stirring. The mixture was filtered and concentrated, the crude was dissolved in AcOEt (15 mL) as organic phase and washed with a saturated solution of NaHCO_3_ (20 mL). The organic layers were dried over Na_2_SO_4_, filtered and the solvent evaporated in vacuo. The crude was purified by silica filtration with DCM/MeOH/NH_3_ in MeOH (9:1:0.2, *v*/*v*/*v*), giving **BOPC1** as yellow oil (33 mg, 35%). The characterization of the compound corresponds to the already published spectra [22].

^1^H-NMR (400 MHz, CDCl_3_): δ 7.25 (ddd, J = 7.4, 4.5, 1.5 Hz, 2H), 7.22–7.15 (m, 3H), 6.30– 6.27 (m, 1H), 6.25 (d, J = 2.2 Hz, 2H), 5.96 (s, 1H), 4.75 (d, J = 7.5 Hz, 1H), 3.87–3.80 (m, 1H), 3.67 (d, J = 3.0 Hz, 6H), 3.62 (d, J = 4.5 Hz, 2H), 3.23 (ddd, J = 16.9, 11.9, 5.1 Hz, 1H), 3.18–3.08 (m, 1H), 2.61 (dq, J = 8.7, 4.9 Hz, 1H), 2.57–2.49 (m, 2H), 2.46–2.34 (m, 2H), 2.15 (dd, J = 13.6, 6.2 Hz, 1H), 2.02–1.83 (m, 4H), 0.78 (d, J = 5.4 Hz, 3H), 0.76 (s, 3H).

^13^C-NMR (101 MHz, CDCl_3_): δ 175.26, 171.13, 161.21, 142.42, 104.53, 99.23, 61.68, 55.31, 52.91, 45.75, 28.78, 26.41, 20.18, 19.97, 18.50. IR (νmax/cm^−1^): 836.9, 910.4, 991, 1061.62, 1156.12, 1203.36, 1607.38, 1746.23, 3002.62, 3027.69, 3381.57. UHPLC-ESI-MS: ABS tR =  1.55 min, 96% pure (λ  =  210 nm), calculated for C_27_H_38_N_3_O_4_ [M + H]^+^ 468.28623, found 468.28601.

### 4.3. Evaluation of (+)-(2S,3S)-BOPC1efficacy in CF Models

Primary human airway epithelial cells (HAECs) derived from cystic fibrosis (CF) donors homozygous for the F508del-CFTR mutation were obtained from the FFC Primary Culture Facility. Cells were expanded and differentiated at the air-liquid interface (ALI) using PneumaCult™-ALI medium (STEMCELL Technologies, Vancouver, BC, Canada) according to the manufacturer’s protocol. Once fully differentiated, HAECs were apically exposed to the HuR inhibitor (+)-(*2S,3S*)-**BOPC1** at final concentrations of 50 µM or 100 µM in PneumaCult™-ALI medium for 24 h. (+)-(*2S,3S*)-**BOPC1** was solubilized in DMSO and diluted in antibiotic-free ALI medium; control cells received vehicle (DMSO) only. *Pseudomonas aeruginosa* PAO1 was cultured overnight in Luria–Bertani (LB) broth at 37 °C with shaking. The bacterial suspension was adjusted to an optical density corresponding to ~1 × 10^7^ colony-forming units (CFU) per well, in phosphate-buffered saline (PBS) solution. Prior to infection, the apical surface of HAECs was gently rinsed with pre-warmed PBS. Cells were then apically infected with the PAO1 inoculum and incubated for 1 h at 37 °C in 5% CO_2_. Following incubation, non-adherent bacteria were removed by washing the apical surface three times with PBS. To recover adherent bacteria, cells were lysed by adding 200 µL of 0.1% Triton X-100 to the apical compartment, followed by vigorous pipetting. Lysates were serially diluted in PBS and plated on LB agar for CFU enumeration after overnight incubation at 37 °C. The percent of bacterial adhesion was calculated relative to the initial inoculum and was normalized to the adhesion of the control (vehicle-treated) strain, set at 100%. Cell viability and morphology were monitored by phase-contrast microscopy throughout the experiment. No signs of cytotoxicity or epithelial detachment were observed at either concentration of (+)-(*2S,3S*)-**BOPC1**. Bacterial adhesion data were analyzed using Ordinary ONE-way ANOVA test. A *p*-value < 0.05 was considered statistically significant. The experiment was performed in triplicate.

## Data Availability

The original contributions presented in this study are included in the article. Further inquiries can be directed to the corresponding authors.
